# Cavum Septi Pellucidi et Vergae in the Pathogenesis of Prenatally Detected Ventriculomegaly

**DOI:** 10.2174/0115734056334652250120092841

**Published:** 2025-03-17

**Authors:** Fatih Ates, Ömer Faruk Topaloglu, Mehmet Sedat Durmaz, Mustafa Koplay

**Affiliations:** 1 Department of Radiology, Selcuk University, Konya, Turkey; 2 Department of Radiology, Serdivan State Hospital\Sakarya, Sakarya, Konya, Turkey

**Keywords:** Fetal MRI, Ultrasonography, Ventriculomegaly, Cavum septi pellucidi, Pathogenesis, Cavum vergae

## Abstract

**Objective:**

The main objective of this work was to investigate the effect of cavum septi pellucidi et vergae (CSPV) on the pathogenesis of ventriculomegaly (VM) cases detected during the fetal period.

**Materials and Methods:**

The fetuses of 515 mothers who applied to the Department of Radiology between October 2011 and December 2022 and who had undergone fetal magnetic resonance imaging (fMRI) were evaluated retrospectively. 152 fetuses with CSPV were included in the study. The fetuses were separated into the following groups: those with right VM (n = 20), those with left VM (n = 56), and those with bilateral VM (n = 44). Fetuses with CSPV, but without VM (n = 32), were included in the study as the control group. For the group with CSPV, lines were drawn to divide the fetal cranium into two symmetrical parts at the interhemispheric line in the axial and coronal planes. The distances from these lines to the lateral leaves of the CSPV were measured. In addition, measurements of the CSPV (anteroposterior, transverse, and high) were taken. An evaluation of whether that was associated with ventricular width or maternal age and gestational week was conducted.

**Results:**

The left ventricular width was significantly higher in cases where the CSPV deviated more to the right, and the right ventricular width was significantly higher in cases where the CSPV deviated more to the left. When the VM rates in the VM group without CSPV and the VM rates in the VM group with CSPV were compared, the VM rates were found to be significantly higher in those with CSPV.

**Conclusion:**

Fetuses with CSPV should be followed up for the possibility of developing VM. However, it should be remembered that VM may be a variation due to CSPV. There is an inverse relationship between the side where CSPV deviates and the side where VM is observed.

## 1 INTRODUCTION

Routine evaluation of the presence of the cavum septi pellucidi (CSP) and cavum septi pellucidi et vergae (CSPV) in the second-trimester obstetric ultrasonography (US) is very important in prenatal follow-up.

A normal CSP or CSPV scan is used as an indicator of the normal development of the midline structures of the cerebral hemispheres [1]. Although some studies describe the relationship of CSP and cavum vergae (CV) with developmental delays and psychiatric disorders in adults, it is generally accepted that the persistence of CSP in postpartum life is a normal variation [2]. It is important to emphasize that CSPV is in direct contact with the subarachnoid space, but it does not communicate with the ventricular system and is not considered part of it. Since these spaces are not lined with ependymal or choroid plexus cells, they do not produce cerebrospinal fluid (CSF) [3].

US may be insufficient for detecting fetal anomalies due to maternal obesity, oligohydramnios, neurological disorders, and other anomalies. Although US is always performed first, in such cases, fetal magnetic resonance imaging (fMRI) should be used to evaluate the midline structures of the brain [4]. fMRIs provide detailed intrauterine imaging of the developing fetus. Fast sequences are required because of fetal movement. fMRIs are most commonly used when US findings are unclear. Using fMRIs, fetal anatomy can be evaluated in detail, including the brain, upper respiratory-digestive system, thorax, pelvis, and abdomen. fMRIs are usually performed during and after the second trimester [2, 4].

In fetuses with isolated moderate ventriculomegaly (VM) (VM with a width of 13–15 mm), additional structural anomalies, especially anomalies, such as supratentorial intracranial haemorrhages, polymicrogyria, and lissencephaly, can be detected through fMRIs, whereas these anomalies are missed in 5.4% of neurosonographic examinations [5, 6].

In this study, the main objective was to investigate the frequency of CSPV in fetuses who were referred for fMRI examinations following the detection of fetal VM during prenatal follow-ups using the US, as well as the frequency of additional cerebral anomalies. In addition, this study aimed to find the relationship among gestational age, gestational week, ventricular width, and the transverse, anteroposterior, and vertical dimensions of CSPV. Moreover, the objective of the study was to determine the asymmetry or symmetry of CSPV and determine its relationship with VM. This study also aimed to investigate whether CSPV is just a variation as a consequence of VM or whether it is one of the etiological causes of VM.

## 2 MATERIALS AND METHODS

### 2.1 Patient Population

This research study was conducted on humans according to the Helsinki Declaration of 1975, as revised in 2013 (http://ethics.iit.edu/ecodes/node/3931). The approval for the study was obtained from the local research ethics committee (approval number: E-70632468-050.01.04-384211). The study retrospectively evaluated 515 fetuses who applied to the radiology department between October 2011 and December 2022, and were found to have fetal anomalies through the obstetric US performed between 16–36 weeks of gestation, and who had undergone fMRIs to search for further anomalies. The study included 152 fetuses with CSPV. Fetal VM and CSPV were present in 120 of these fetuses. Ventricular width was within normal limits in 32 fetuses. Of the 120 fetuses, 20 had isolated right VM, 56 had isolated left VM, and 44 had bilateral VM. The selection of the patient population was presented in Fig. (**1**). For a comparison with the group without CSPV, a group with VM, but not CSPV, and a group of fetuses that had fMRIs for VM, but received negative results, were also established. Seventy-two of the 515 fetuses had VM, but not CSPV. There were also negative findings in 71 fetuses without CSPV who underwent fMRI for VM (Table **1**). The group with CSPV and VM was compared with the group without CSPV, but with VM. The patient selection scheme is displayed in Fig. (**1**). Differences in age, pregnancy, and measurement values according to the VM aspect were also evaluated.

### 2.2 MRI Protocol

An abdominal coil was used in the MRI (Magnetom Aera, Siemens Healthcare, Erlangen, Germany) device with 1.5 Tesla magnetic field strength. Before examination, a fasting period of 6–8 hours was requested from pregnant women. After a routine MRI examination of relevant regions of the fetus according to clinical information, the fetal central nervous system (CNS) was scanned. Fetal MRI parameters are described in Table **2**.

No sedation medication or contrast material method was used. The mother was not told to hold her breath. Two or three trials were performed for each sequence in cases where optimal images could not be obtained due to fetal movement during sequences taken. Despite this, shooting was terminated in cases where fetal movement continued. The total acquisition time of MRI sequences of the fetal cranium did not exceed 15 minutes.

### 2.3 Analysis of Images and Measurements

**Fig. (2):**
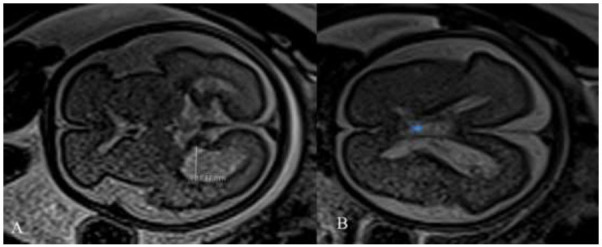
In the axial T2 HASTE images of the fetal cranium, the lateral ventricle diameter was measured as 11.6 mm at the level of the atrium (**A**). CSPV variation (**B**) is observed in the inferior section of the same patient (Blue star; CSPV: Cavum septum pellucidum et Vergae).

**Fig. (3):**
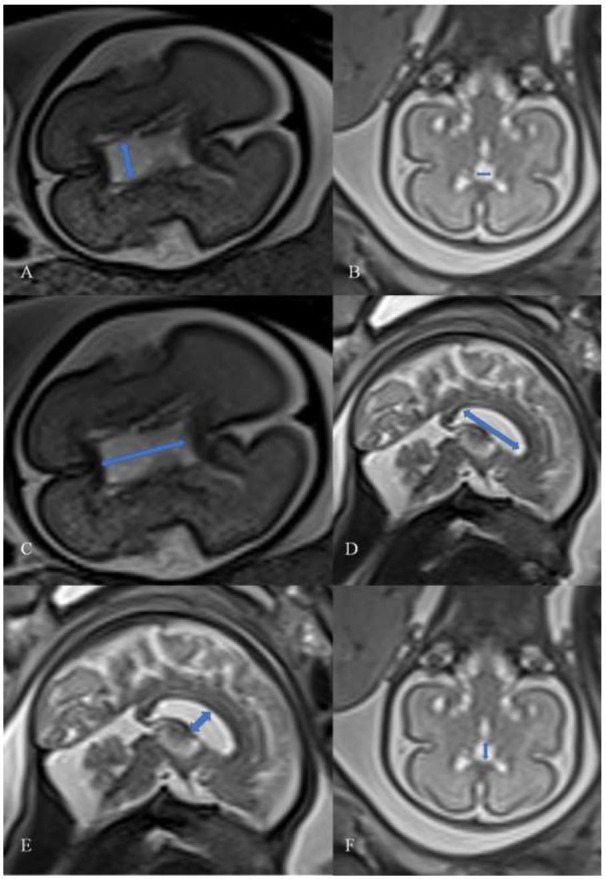
Transverse (**A**, **B**), sagittal (**C**, **D**), and vertical (**E**, **F**) measurements of CSPV on axial, coronal, and sagittal plans. Each measurement was confirmed with other plans, as demonstrated in the figure (CSPV: Cavum septum pellucidum et Vergae).

All images were evaluated by a radiologist with six years of experience in fetal radiological imaging. Last menstrual time (LMT) and US biometry measurements were used for fetal age determination. Biometry measurements were accepted for fetal age determination in pregnant women with inconsistency between LMT and US measurements. CSPV variation and VM (isolated right, isolated left, bilateral) were included in the study. Fetal lateral ventricles were measured at the level of the atrium in the axial sections (Fig. **2**). The diameters of the detected CSPV variations were measured in transverse (TR), anteroposterior (AP), and height (H) in two planes (Fig. **3**), and it was confirmed that there was no difference between the sections. Such an application was made to minimise the error in the measurement. The relationship among AP, TR, and H dimensions of total variation and gestational week, age, and ventricular width, was evaluated.

**Fig. (4):**
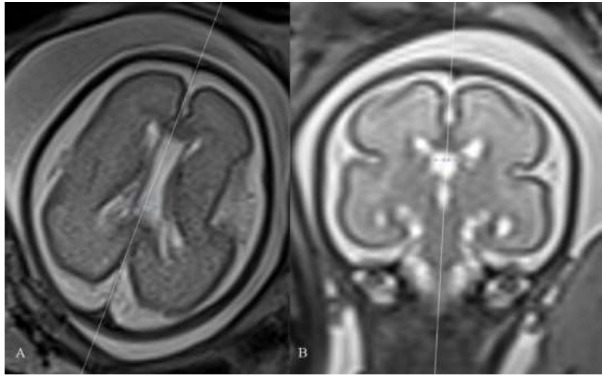
In the axial (**A**) and coronal (**B**) plans of T2W images, the fetal cranium was divided into symmetrical equal parts by the line traversing the interhemispheric line. To avoid the effect of deviations, a line dividing the fetal cranium into two equal parts was drawn from the interhemispheric line to the level of the mesencephalon and pons in the coronal section. The distances from this line to the lateral leaves of the CSPV were noted (T2W: T2 weighted image; CSPV: Cavum septum pellucidum et Vergae).

In addition, to investigate whether there was a right or left deviation of the CSPV relative to the midline, the line dividing the fetal cranium into two symmetrical parts was drawn in the axial section, starting from the anterior interhemispheric fissure level and extending to the posterior interhemispheric fissure level. To confirm the right or left deviation of CSPV in the coronal plane due to possible hemispheric fissures or deviations, the line extending vertically from the interhemispheric area to the level of mesencephalon and pons and dividing the fetal cranium into two symmetrical parts was drawn. Thus, an attempt was made to avoid the effect of possible deviations. The distance from these lines to the lateral leaves of the CSPV was measured (Fig. **4**). Measurement could not be made in some limited patients due to asymmetry, fetal movement, and fetal inappropriate position. These fetuses were excluded from the study. fMRI prediagnoses and post-examination diagnoses are illustrated in Table **3**.

### 2.4 Statistical Analysis

Parametric tests were used without a normality test due to the compatibility of the central limit theorem [7]. In the data analysis, mean, standard deviation, and minimum and maximum values of features were used while setting the data in a continuous structure. One-way ANOVA test statistic was used to compare the mean of more than two groups of measurements. If a difference was detected with ANOVA, a post-hoc test was performed using Tukey’s statistic. Pearson’s correlation coefficient was used when examining relationships between continuous measurements. The correlation coefficient (*r*) degree of the relationship was evaluated as *r* < 0.29 (very weak), 0.30 ≤ *r* < 0.50 (weak), 0.5 ≤ *r* < 0.70 (moderate), 0.70 ≤ *r* < 0.90 (high), and *r* ≥ 0.90 (very high). The statistical significance level of data was taken as *p* < 0.05. IBM SPSS version 25 software and the MedCalc statistical package program were used to evaluate the data.

## 3 RESULTS

The mean age of pregnancy in the study was 28.3 ± 5.3 (17–42 weeks). There was no difference between mean ages according to the VM direction (*p* > 0.05). The mean age was 27.4 ± 4.9 years in the right VM group, 29.3 ± 4.7 years in the left VM group, 27.8 ± 5.4 years in the bilateral VM group, and 27.5 ± 6.1 years in the control group.

VM rates were significantly higher in the group with CSPV than the group without CSPV, but with VM (*p* < 0.005; Table **1**).

Furthermore, 118 of 152 fetuses were between 23 and 30 weeks of gestation. Gestational week was 27.6 ± 3.4 in the right VM group, 27.5 ± 3.1 in the left VM group, 27.8 ± 5.4 in the bilateral VM group, and 27.3 ± 4.3 in the control group. No statistically significant difference was observed between the mean weeks of gestation according to VM direction (*p* > 0.05).

According to VM direction, there was a significant difference in the mean measurement of the ventricle (mm) between the control group and the right, left, and bilateral VM groups (p* < 0.05). Mean measurement was 5.94 ± 1.63 mm in the control group, 11.81 ± 2.45 mm in the right VM, 10.73 ± 1.69 mm in the left VM, and 10.78 ± 1.67 mm in the bilateral VM group. Mean measurements in the control group were significantly lower than in other groups.

A significant difference was observed between the control group and the bilateral VM group in the mean of CSPV TR measurements (p* < 0.05). TR measurements were 4.97 ± 1.81 mm in the control group, 5.11 ± 1.32 mm in the right VM group, 4.58 ± 1.26 mm in the left VM group, and 3.97 ± 1.58 mm in the bilateral VM group. The mean TR measurement in the control group was significantly higher than the bilateral VM group.

There was a significant difference in the right variation measurement averages between the control group and the VM right group, and between the left group and the bilateral and right VM groups (p* < 0.05). Measurements of deviation to the right were 2.51 ± 0.92 mm in the control group, 1.81 ± 0.87 mm in the right VM group, 3.01 ± 0.99 mm in the left VM group, and 1.98 ± 0.83 mm in the bilateral group. There was also a significant difference between left and bilateral VM, the right group and the left and bilateral groups, and the left group and the bilateral and control groups in the mean of left variation measurements (p* <0.05). Deviation measurement to the left was 2.47 ± 0.99 mm in the control group, 3.33 ± 0.95 mm in the right VM group, 1.59 ± 0.58 mm in the left VM group, and 2.03 ± 0.84 mm in the bilateral VM group. No significant difference was observed between the AP and H measurement averages according to VM direction (*p* > 0.05) (Table **4**).

No significant relationship was observed between maternal age and total CSPV measurements (*p* > 0.05). A weak positive and significant relationship was observed between the week of gestation and CSPV, AP, and H measurements (*p* < 0.05). A very weak positive and significant correlation was found between ventricular width and CSPV and H measurements (*p*<0.05). There was a very weak negative significant relationship between ventricular width and CSPV leaf deviation to the right (*p* < 0.05; Table **5**).

The relationship between AP, TR, and H dimensions of CSPV in the right VM group and gestational week, age, and ventricular width was evaluated. No significant relationship was observed between maternal age and gestational week and CSPV measurements (*p* > 0.05). A moderately positive and significant relationship was observed between ventricular width and CSPV H measurements (*p* < 0.05). Likewise, the relationship in the left VM group was evaluated; no significant correlation was found in this group (*p* > 0.05). In the bilateral VM group, no significant relationship was observed between maternal age and CSPV measurements (*p* > 0.05). A weak positive and significant relationship was observed between gestational week and CSPV H measurements (*p* < 0.05). There was also a weak positive and significant correlation between ventricular width and CSPV H measurements (*p* < 0.05). However, in the bilateral VM group, no significant relationship was observed between maternal age and gestational week and CSPV measurements (*p* > 0.05). We found a weak positive correlation between ventricular width and CSPV TR, a moderate correlation with CSPV AP, and a weak positive correlation with CSPV leaf deviation to the right (*p* < 0.05).

## 4 DISCUSSION

The persistence of CSPV may be associated with conditions, such as chronic brain trauma, post-traumatic stress disorder, schizophrenia, dementia, headache, intracranial hypertension, and personality changes in adults [8-11]. Some studies have reported the size and frequency of CSPV to be higher in patients with schizophrenia, alcoholism, and pre-existing head trauma [12]. CSP and CV enlargement may be associated with hydrocephalus, midgut malrotation, volvulus, and VM [13-15].

In fetal VM, a comprehensive investigation should be performed in search of other intracranial or extracranial abnormalities because truly isolated and mild VM has a much different prognosis than VM with other findings. Newer literature should be consulted during investigations into this, especially since researchers conducting studies on this matter in the past did not have access to today's techniques and technology. In this regard, a literature study [16] evaluated 34 cases of mild VM, and another study [17] assessed 30 cases. The first study [16] found that one-third of the mild VM cases improved in the postnatal control group, and 50% of them returned to normal ventricle diameter spontaneously. The second study [17] reported that 21 out of 30 mild VM cases were isolated, of which only four had a neurological abnormality after birth [16, 17]. In another study [18], which included 176 cases of fetal VM, ventricular enlargement was classified as mild (10–12.5 mm), moderate (12.5–15 mm), or severe (greater than 15 mm). In those with isolated mild VM, the chance of good survival (up to 24 months) was 97.7%-80% in the isolated moderate group and 33% in the isolated severe group. However, there is no consensus on this classification, as some studies have classified all cases of VM with atrial measurements between 10 and 15 mm as mild [19, 20]. For this reason, we did not classify VM in our study. However, truly isolated VM has an excellent prognosis, especially when measurement is between 10 and 12.5 mm [16-18]. Therefore, it is necessary to be meticulous in VM diagnosis. Studies have been conducted on the prenatal or postnatal period of VM and its etiologic factors [21-23]. To the best of our knowledge, no study has investigated CSPV in the pathogenesis of VM in the prenatal period.

The CSP originates from an embryological structure known as the “lamina reuniens”, formed by the thickening of the upper end of the lamina terminalis at around 7 weeks of gestational age. Beginning at 9 weeks of gestational age, the anterior commissure and the hippocampal-septal fibers forming the forniceal columns start to develop within the lamina reuniens. By the 11^th^ week, the hippocampal-septal fibers begin to cross the midline within the posterior lamina reuniens, giving rise to the hippocampal commissure. At the 12^th^ week, the interhemispheric fissure deepens and divides the lamina reuniens, and its lateral walls form the leaves of the CSP. In our study, pregnant patients were between 17 and 42 weeks of gestation. Since fetal anomalies are typically detected during the second trimester, no fetal MRI scans were performed during the first trimester. Therefore, we were unable to evaluate the early embryological development of the CSP and CSPV cases [2, 24].

CSPV is a closed cavity filled with CSF that is not connected to a ventricular system or subarachnoid space. However, in the literature [25], it has been reported that CSP and CV can be classified as communicating or non-communicating by connecting lateral ventricles with fenestrations. In addition, endoscopic fenestration is applied to lateral ventricle walls or the third ventricle in enlarged CSPV cases [26, 27]. According to the literature, CSPV plays a role in the pathogenesis of various cognitive and psychiatric disorders in adults. A study [28] reported a case of late-onset catatonia in a 66-year-old female patient with CSPV, noting that midline developmental anomalies may be a factor in psychotic disorders, even in the elderly. While another study [11] reported that CSPV may be the main factor behind 64-year-old male patient's new-onset hallucinations and schizophrenia-like psychotic disorder. In various adult patient studies, it has been stated that enlarged CSPV is frequently seen in psychiatric patients and is accepted as evidence of impaired midline development of the brain, especially the limbic system [29]. In a study that systematically evaluated 1,070 children aged 6–10 years [8], in cases with enlarged CSP, there was a larger corpus callosum and thalamus, as well as more white matter overall compared to total brain volume. However, the study did not find significant cognitive function or emotional or behavioural impairment in children examined. In addition, in this study, volumes of the lateral ventricles of children with enlarged CSP were lower than in the control group. In contrast to this study, we found a significant relationship between CSPV and VM. We measured the distance of CSPV leaves to the imaginary line drawn in the midline of the interhemispheric plane. Our aim was to determine whether CSPV asymmetry affects VM. There was a significant difference between the left VM group and the CSPV right deviation (p* < 0.05). In addition, we found a significant difference between the opposite right VM and CSPV left deviation (p* < 0.05). Here, we obtained the following result: as the distance from the CSPV midline to the right leaf increases, the frequency of the left VM increases, and as the distance from the CSPV midline to the left leaf increases, the frequency of the right VM increases. Fenestrations to the lateral ventricle, which cannot be seen on an fMRI, but may be microscopic, could be responsible for this situation. In addition, the close proximity of CSPV with the foramen of Monro is anatomically known. We believe that the probability of compression on interventricular foramen and the possibility of lateral VM increase as CSPV sizes increase. In a study reported in 1993 [30], five cases of persistent hydrocephalus and related symptoms (intermittent postural headache and loss of postural consciousness) due to direct compression of the CSP were reported. The study reported that this pathological condition caused ventricular expansion, and symptoms developed, possibly due to sudden transient occlusion of the foramen of Monro. One of these five cases was reported to have taken place in a six-month-old infant. The others were adult cases. As mentioned above, no study has investigated the relationship between CSPV and VM in the prenatal period.

In our study, the indication for fMRI was VM in 114 (75%) of 152 fetuses. This was followed by MCM, with 16 fetuses (10.52%). Fetal cerebral VM was the most common diagnosis in our study group, with 121 fetuses (79.6%) diagnosed after an fMRI. However, fMRI results were completely normal in 10 fetuses (10.52%). This was followed by MCM, with seven fetuses (4.6%). Compared to the literature, the most common fMRI indication is CNS malformations, including VM [31, 32]. Our aim was not to compare the pre-fMRI diagnoses with post-examination findings. However, we wanted to understand the indications and findings that were the subject of this study. We examined the effect of CSPV on the pathogenesis of VM. For this purpose, we felt the need to contact fetuses who do not have CSPV, but have VM. In this group, there were 72 fetuses with VM (36 left VM, 19 right VM, 17 bilateral VM). In this group, the prediagnoses of 60 fetuses were VM. We found the mean ventricular width of 10.68 mm in the group with isolated right VM, 10.62 mm in the group with isolated left VM, and 11.6 mm in the group with bilateral VM. In total (with and without CSPV group), we observed 92 fetuses with isolated left VM (with CSPV: 56; without CSPV group: 36), 39 fetuses with isolated right VM (with CSPV: 20; without CSPV: 19), and 61 fetuses with bilateral VM (with CSPV: 44; without CSPV: 17). Overall, 192 fetuses with VM were detected. When this number was compared with the total number (515 fetuses), the VM rate was 37.28%. In addition, when the group with CSPV and VM and the group without CSPV, but with VM, were compared, VM rates were significantly higher in the group with CSPV (*p* < 0.001). With this information, we can propose CSPV as one of the factors in the pathogenesis of VM.

We found a significant difference in TR measurements between the control group and the bilateral VM group (p* < 0.05). The mean measurement was higher in the control group than in the bilateral group. In our opinion, this situation is because, in bilateral VM, the enlarged lateral ventricles may partially compress the CSPV leaves in the midline, resulting in a lower CSPV TR diameter because the ventricle width was significantly higher in the bilateral VM group compared to the control group. This may be partially due to increased CSF circulation. In addition, we found that the H and AP lengths of CSPV increased as the gestational week progressed. This may be due to fetal cerebrum and fetal calvaria structures that develop and grow as pregnancy progresses. The CSPV TR diameter was significantly lower in the bilateral VM group than in the control group. In this case, it may be because as the TR diameter decreases, the ventricle expands in other directions. As a result of all these aspects, there are various publications focused on fetal VM and CSPV [8, 22, 33-36]. However, to the best of our knowledge, a study evaluating these two entities together is not available in the current literature.

### 4.1 Limitations

We did not evaluate fetuses in postnatal follow-up. For this reason, we could not compare VM and CSPV dimensions in prenatal and postnatal periods. In addition, we did not evaluate whether any neurological symptoms would develop in these fetuses during the postnatal period. Because most of these patients were followed in other centres in the postnatal period, this process is the subject of other prospective large-scale controlled studies.

## CONCLUSION

Fetuses with CSPV should be evaluated in terms of the possibility of developing VM. In the presence of CSPV, the possibility and direction of VM development in fetuses can be predicted according to the right or left deviation. CSPV may also be a variation that may accompany VM. More extensive and comprehensive studies covering prenatal and postnatal periods should be conducted in this regard.

## Figures and Tables

**Fig. (1):**
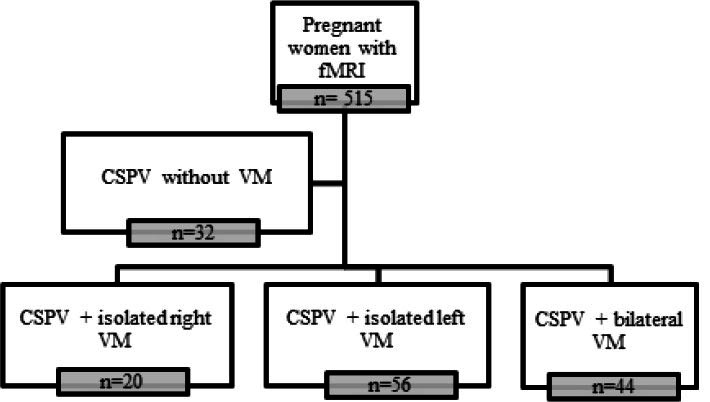
Patient population. **Note:** (fMRI: Fetal magnetic resonance imaging; CSPV: Cavum septum pellucidum et Vergae; VM: Ventriculomegaly).

**Table 1:** Evaluation of VM and CSPV association.

-	**CSPV (+)**	**CSPV (-)**	***p*-value**
**VM (+)**	120 (%78,9)	72 (%50,3)	*p*<0.001
**VM (-)**	32 (%21,1)	71 (%49,7)	*p*<0.001
**Total**	152	143	-

**Note:** *p* < 0.001 according to Pearson’s chi-square test; CSPV: Cavum septum pellucidum et Vergae.

**Table 2:** Parameters of fetal MRI sequences.

**Sequence**	**TR** **(ms)**	**TE (ms)**	**FOV (mm)**	**Slice Thickness (mm)**	**Voxel Dimensions (mm)**	**Slices**
Fetal whole body	-	-	-	-	-	-
T2- HASTE axial	1010	95	250	5	1x1x5	30
T2- HASTE coronal	1050	94	250	4	1,3x1,3x4	30
T2- HASTE sagittal	1400	94	250	4	1,3x1,3x4	20
Fetal cranium	-	-	-	-	-	-
T2- HASTE axial	1010	94	230	3	1,2x1,2x3	27
T2- HASTE coronal	1010	94	230	3	1,2x1,2x3	30
T2- HASTE sagittal	1100	94	220	3	1,1x1,1x3	20
SWI	44	35	230	2,3	0,9x0,9x2,3	30
T1-2D GRE axial	108	4,76	280	3	1,1x1,1x3	20
**DWI**	**6600**	**91**	**230**	**3**	**0,6x0,6x3**	**28**

**Abbreviations: **TR: Repetition time; TE: Echo time; FOV: Field-of-view; HASTE: Half Fourier acquired single shot turbo spin echo; SWI: Susceptibility weighted image; T1-2D GRE: T1 two-dimensional gradient echo; DWI: Diffusion weighted images.

**Table 3:** Indications of fMRI in cases with CSPV and diagnoses after fMRI.

**Prediagnosis**	**n=152**	**%**	**Diagnose after fMRI**	**%**	-
VM	114	75	121	79,6	-
Normal	-	-	16	10,52	-
MCM	16	10,52	7	4,6	-
Cleft palate-lip	5	3,29	1	0,66	-
CCA/CCD	4	2,63	-	-	-
Inferior Vermian agenesis	3	1,97	1	0,66	-
Hydrocephalus	3	1,97	-	-	-
Microcephaly	2	1,32	-	-	-
Twin, ex of sibling	1	0,66	-	-	-
Oral cavity mass	1	0,66	1	0,66	-
Lissencephaly	1	0,66	2	1,32	-
Diastomatomyeli	1	0,66	1	0,66	-
Micrognati	-	-	1	0,66	-
Holoprosencephaly	-	-	1	0,66	-
Arachnoid cyst	1	0,66	-	-	-

**Abbreviations: **fMRI: Fetal magnetic resonance imaging; CSPV: Cavum septum pellucidum et Vergae; VM: Ventriculomegaly; MCM: Mega cisterna magna; CCA/CCD: Corpus callosum agenesis/ dysgenesis.

**Table 4:** Evaluation of difference according to VM aspect in age, gestational age, and measurement values.

**Ventriculomegaly**		**Total (n=152)**	**Right (n=20)**	**Left (n=56)**	**Bilateral (n=44)**	**Control (n=32)**	-	-
		**x̄ ±S D**	**x̄ ±S D**	**x̄ ±S D**	**x̄ ±S D**	**x̄ ± SD**	***p*-value**	*p*-value* **1 *vs*. 2 1 *vs*. 3 1 *vs*. 4** **2 *vs*. 3 2 *vs*. 4 3 *vs*.4**
**Measurements**		**Min-Max**	**Min-Max**	**Min-Max**	**Min-Max**	**Min-Max**
**Age**		28,3±5,3	27,4±4,9	29,3±4,7	27,8±5,4	27,5±6,1	0,34	-
		17-42	17-37	19-39	17-38	19-42	-	-
**Gestational week**		27,2±3,321-37	27,6±3,417-42	27,5±3,122-34	26,7±2,921-36	27,3±4,320-34	0,63	-
**Ventricular width**		9,88±2,723,6-18	11,81±2,458-18	10,73±1,697,5-14	10,78±1,678-15	5,94±1,633,6-9,5	**<0,001**	0,11,0,15**,<0,001**0,99,**<0,001,<0,001**
**CSPV TR**		4,58±1,541,6-8,7	5,11±1,323,1-6,9	4,58±1,262-6,7	3,97±1,581,6-8,4	4,97±1,811,6-8,7	**0,009**	0,53,**0,03**,0,990,19,0,63,**0,02**
**CSPV AP**		26,91±4,0815,4-37,3	28,24±5,4817,2-37,3	26,73±3,3318,8-33,1	27,32±3,1619,8-34	25,84±5,1715,4-34,6	0,18	-
**CSPV H**		7,16±1,743,1-13	7,45±1,844,2-10,7	7,14±1,583,1-11,7	7,52±1,655,2-13	6,52±1,923,9-10,4	0,08	-
**CSPV, Deviation to right**		2,45±1,030,8-5,2	1,81±0,870,8-3,9	3,01±0,991-5,2	1,98±0,830,8-4,3	2,51±0,920,8-4,8	**<0,001**	**<0,001**,0,9,0,**04<0,001**,0,08,0,07
**CSPV, Deviation to left**		2,13±0,980,8-5,2	3,33±0,950,9-5,2	1,59±0,580,8-3	2,03±0,840,8-4,2	2,47±0,990,8-5	**<0,001**	**<0,001,<0,001,0,0020,04,<0,001**,0,1

**Abbreviations: **CSPV: Cavum septum pellucidum et Vergae; TR: Transverse; AP: Anteroposterior; H: Height; VM: Ventriculomegaly.

**Table 5:** The relationship between AP, TR, and H dimensions of total variation and gestational week, age, and ventricular width.

**N:152**	-	Age	Gestational week	Ventricular widht
CSPV TR	**r**	-0,1	0,13	-0,20
	**p**	0,33	0,11	0,02
CSPV AP	**r**	-0,04	0,35	0,20
	**p**	0,96	**<0,001**	0,02
CSPV H	**r**	-0,07	0,25	0,35
	**p**	0,44	**0,007**	**<0,001**
CSPV, Deviation to right	**r**	-0,03	0,1	-0,17
	**p**	0,68	0,22	**0,04**
CSPV, Deviation to left	**r**	-0,10	0,1	-0,11
	**p**	0,22	0,22	0,19

**Note: **CSPV: Cavum septum pellucidum et Vergae; TR: Transverse; AP: Anteroposterior; H: Height; *r*: Correlation coefficient degree of relationship; *r* < 0.29 very weak, 0.30 ≤ *r* < 0.50 weak, 0.5 ≤ *r* < 0.70 moderate, 0.70 ≤ *r* < 0.90 high, *r* ≥ 0.90 very high. The statistical significance level of the data was taken as *p* < 0.05.

## Data Availability

The data and supportive information are available within the article.

## LIST OF ABBREVIATIONS

CSPV: Cavum Septi Pellucid et Vergae
VM: Ventriculomegaly
fMRI: Fetal Magnetic Resonance Imaging
CSP: Cavum Speti Pellucidi
US: Ultrasonography
CV: Cavum Vergae
CSF: Cerebsospinal Fluid
CNS: Central Nervous System
TR: Repetition time
TE: Echo time
FOV: Field-of-view
HASTE: Half Fourier acquired single shot turbo spin echo
SWI: Susceptibility weighted image;
T1-2D GRE: T1 two-dimensional gradient echo
DWI: Diffusion weighted images
LMT: Last Menstrual Time
TR: Transverse
AP: Anteroposterior
H: Height
CCA: Corpus Callosum agenesis
CCD: Corpus Callosum dysgenesis

## STANDARDS OF REPORTING

STROBE guidelines were followed.
